# TeloBase: a community-curated database of telomere sequences across the tree of life

**DOI:** 10.1093/nar/gkad672

**Published:** 2023-08-21

**Authors:** Martin Lyčka, Michal Bubeník, Michal Závodník, Vratislav Peska, Petr Fajkus, Martin Demko, Jiří Fajkus, Miloslava Fojtová

**Affiliations:** Mendel Centre for Plant Genomics and Proteomics, Central European Institute of Technology (CEITEC), Masaryk University, BrnoCZ-62500, Czech Republic; National Centre for Biomolecular Research, Faculty of Science, Masaryk University, BrnoCZ-62500, Czech Republic; National Centre for Biomolecular Research, Faculty of Science, Masaryk University, BrnoCZ-62500, Czech Republic; Mendel Centre for Plant Genomics and Proteomics, Central European Institute of Technology (CEITEC), Masaryk University, BrnoCZ-62500, Czech Republic; National Centre for Biomolecular Research, Faculty of Science, Masaryk University, BrnoCZ-62500, Czech Republic; Department of Cell Biology and Radiobiology, Institute of Biophysics, Academy of Sciences of the Czech Republic, BrnoCZ-61200, Czech Republic; Mendel Centre for Plant Genomics and Proteomics, Central European Institute of Technology (CEITEC), Masaryk University, BrnoCZ-62500, Czech Republic; Department of Cell Biology and Radiobiology, Institute of Biophysics, Academy of Sciences of the Czech Republic, BrnoCZ-61200, Czech Republic; Core Facility Bioinformatics, Central European Institute of Technology (CEITEC), Masaryk University, BrnoCZ-62500, Czech Republic; Faculty of Informatics, Masaryk University, BrnoCZ-62500, Czech Republic; Mendel Centre for Plant Genomics and Proteomics, Central European Institute of Technology (CEITEC), Masaryk University, BrnoCZ-62500, Czech Republic; National Centre for Biomolecular Research, Faculty of Science, Masaryk University, BrnoCZ-62500, Czech Republic; Department of Cell Biology and Radiobiology, Institute of Biophysics, Academy of Sciences of the Czech Republic, BrnoCZ-61200, Czech Republic; Mendel Centre for Plant Genomics and Proteomics, Central European Institute of Technology (CEITEC), Masaryk University, BrnoCZ-62500, Czech Republic; National Centre for Biomolecular Research, Faculty of Science, Masaryk University, BrnoCZ-62500, Czech Republic

## Abstract

Discoveries over the recent decade have demonstrated the unexpected diversity of telomere DNA motifs in nature. However, currently available resources, ‘Telomerase database’ and ‘Plant rDNA database’, contain just fragments of all relevant literature published over decades of telomere research as they have a different primary focus and limited updates. To fill this gap, we gathered data about telomere DNA sequences from a thorough literature screen as well as by analysing publicly available NGS data, and we created TeloBase (http://cfb.ceitec.muni.cz/telobase/) as a comprehensive database of information about telomere motif diversity. TeloBase is supplemented by internal taxonomy utilizing popular on-line taxonomic resources that enables in-house data filtration and graphical visualisation of telomere DNA evolutionary dynamics in the form of heat tree plots. TeloBase avoids overreliance on administrators for future data updates by having a simple form and community-curation system for application and approval, respectively, of new telomere sequences by users, which should ensure timeliness of the database and topicality. To demonstrate TeloBase utility, we examined telomere motif diversity in species from the fungal genus *Aspergillus*, and discovered (TTTATTAGGG)_*n*_ sequence as a putative telomere motif in the plant family Chrysobalanaceae. This was bioinformatically confirmed by analysing template regions of identified telomerase RNAs.

## Graphical Abstract

**Graphical Abstract:**
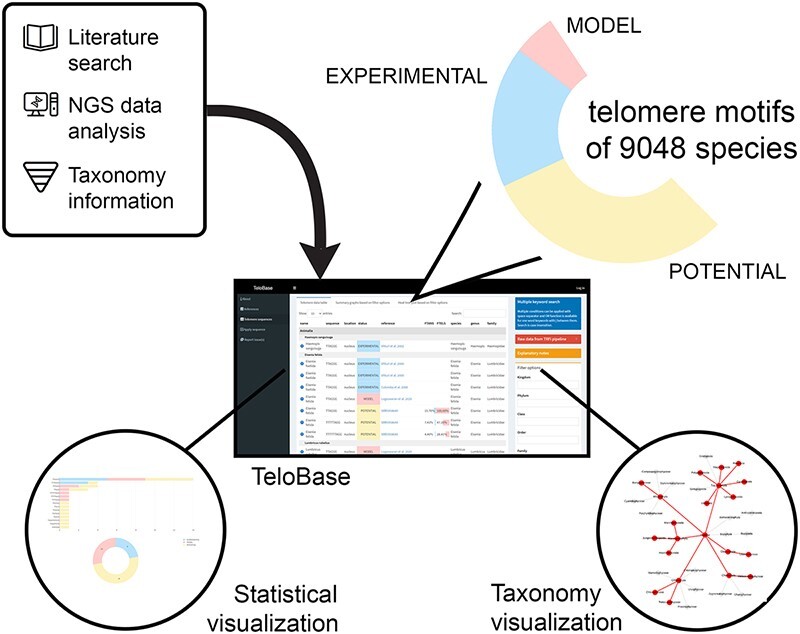
TeloBase content and function summary.

## Introduction

Linear chromosomes pose a distinct challenge for genome stability and replication. Eukaryotes overcome this issue by capping the ends of chromosomes with specialized nucleoprotein structures – telomeres. Telomeres are complex nucleoprotein structures that distinguish the natural ends of chromosomes from DNA breaks, and protect coding regions of the genome from loss due to the incomplete replication of chromosome ends. This replicative shortening of telomeres can be counteracted by the activity of the telomerase. This enzyme is able to add telomere repeats to the 3′ ends of telomeres. The information about the telomere repeat sequence is stored in the template region of telomerase RNA (TR), and the telomere is elongated by the reverse transcriptase activity of the telomerase protein subunit. The activity of telomerase is strictly regulated during development and is limited to tissues with high proliferative capacity in both mammals and plants. Telomere DNA usually consists of short tandemly repeated motifs that, in a broader sense, follow the consensus (T_*x*_A_*y*_G_*z*_)_*n*_ (reviewed in (1)). Nevertheless, the true diversity of these motifs is more multifarious than originally thought.

Over 40 years have passed since the discovery of the first telomere motif (TTGGGG)_*n*_ in the ciliate *Tetrahymena thermophila* (2). The following two decades showed the first glimpses of telomere sequence diversity in yeasts (3,4) but also established several simplified views on the ‘canonical’ telomere motifs in major taxonomic groups. For example, (i) (TTTAGGG)_*n*_ motif was discovered in *Arabidopsis thaliana* (5) and later works confirmed its presence in many other plant species (6,7); (ii) (TTAGGG)_*n*_ repeat was characterised in the order Chordata and other major groups within the kingdom Animalia (8–11); (iii) (TTAGG)_*n*_ terminal sequence was confirmed at chromosomes of many arthropods (9,12); (iv) (TTAGGC)_n_ motif was described in *Ascaris lumbricoides* (13) and is considered the ‘canonical’ telomere in nematodes (14). While we are still waiting for the first ‘non-canonical’ telomere motif in vertebrates and nematodes, the situation in other taxa turned out to be more diverse. In plants (reviewed in (1)), the ‘vertebrate-type’ telomere sequence was discovered in many vascular plants (15–18) as well as in algae, which suggests that (TTAGGG)_*n*_ is the ancestral plant telomere motif (19). A completely novel plant telomere repeat (CTCGGTTATGGG)_*n*_ appeared in the genus *Allium* (20) belonging to the order Asparagales. Other atypical telomere sequences were found in *Cestrum elegans*, from the order Solanales possessing the (TTTTTTAGGG)_*n*_ motif (21), in the order Lamiales where telomeres of some species of the genus *Genlisea* switched to the (TTTCAGG)_*n*_ repeat or its variant (TTCAGG)_n_ while others retained the ‘plant-type’ sequence (22,23). Unusual telomere motifs in insects (reviewed in (24)) were established based on the discovery of retrotransposon-like telomeres in the genus *Drosophila* (25–27) and telomeres consisting of long tandem repeats in the genus *Chironomus* (28–31). Modifications of the ‘insect-like’ motif as (TCAGG)_*n*_ or (TTGGG)_*n*_ in beetles (32,33) and reappearance of the ‘vertebrate-like’ (TTAGGG)_*n*_ sequence in ants of the genus *Myrmecia* were then found (34). Recent discoveries of the (TTATTGGG)_*n*_ motif in parasitoid wasps (35,36) or (TTAGGTTGGGG)_*n*_ in representatives of the genus *Bombus* (36,37) further demonstrate how extensively telomere motifs have been transformed in the course of evolution. The latest findings were possible mainly due to major advances in sequencing technologies that could be employed for the search for telomere repeat candidates (33,35–39). Bioinformatic data mining was successfully applied in confirming the telomere sequences described above, and corresponding TRs templating their synthesis in the genus *Allium* (20), as well as ‘vertebrate-like’ telomeres in seagrass *Zostera marina* (40) or (TTAGGTTGGGG)_*n*_ in *Bombus terrestris* (36).

Currently, there are two databases, Telomerase database (41) and Plant rDNA database (42), harbouring some information about the diversity of telomere motifs. The Plant rDNA database, as the name suggests, focuses primarily on rDNA, its chromosome position and arrangement (42). However, authors in its third version implemented information about the plant telomere motifs in selected species, citing 81 literature sources (43). In comparison, Telomerase database contains information from other taxa besides the plant kingdom, yet the extent of literature sources is even more limited, citing only 61 publications with the latest one published more than a decade ago. This comes as no surprise as the main focal point of the resource is the telomerase enzyme (41). Therefore, it is apparent that there are currently no databases extensively covering decades of accumulated knowledge about telomere motifs in nature.

To fill this gap, we carried out an exhaustive literature review supplemented by an additional search for potential telomere sequences from publicly available next generation sequencing (NGS) data allocated in the NCBI database, in order to expand the number of species with currently known/predicted telomere motifs. To accommodate all gathered data, we created TeloBase (http://cfb.ceitec.muni.cz/telobase), a database that provides not only interactive manipulation and visualisation of the data in the form of graphs or heat tree plots, but also allows user-based curation. We believe that interactivity, curation without dependence on administrators and future updates implementing additional features will lead to the building of an active scientific community around TeloBase that will ensure its longevity and topicality.

## Materials and methods

### Data collection

#### Literature search

Web search engine Google Scholar was utilized in collecting scholarly literature related to telomere sequences published between years 1978 and 2022 (the last search conducted in September 2022) using following keywords: ‘telomere sequence’ OR ‘telomeric sequence’; ‘telomere probe’ OR ‘telomeric probe’; ‘telomere repeat’; telomeric repeat’. Each keyword was used to look for articles published within one specific year apart from years 1978 to 1990 and 1991 to 1995 that were united as none of the keywords reached over 1000 hit limit set by Google Scholar (https://scholar.google.com/) within these selected timeframes. Non-peer-reviewed publications were excluded with one exception (37) as this article focused directly on telomere diversity. Articles with self-contradictory or problematic data (e.g. inconsistent sequence of the telomere motif throughout the article, obvious misspellings in the reported telomere probe for FISH analysis) were excluded. Additionally, reviews were used to assess the completeness of the search as well as to find articles that might have been missed in the literature screen.

#### NGS data collection

The raw data in fasta format were collected in 2018 from Sequence Read Archive (44) using Run selector tool with the following strategy (‘Eukaryota’[Organism] OR eukaryotes[All Fields]) AND (cluster_public[prop] AND ‘biomol dna’[Properties] AND ‘strategy wgs’[Properties]) AND random[Selection]. The full list of corresponding datasets was processed by extracting the first available dataset per each species. The data download, up to 10 mil reads per dataset, was performed by fastq-dump tool in the recommended package sratoolkit.2.9.2-ubuntu64 (NCBI, unpublished, https://github.com/ncbi/sra-tools) (example: $./fastq-dump -X 10000000 -Z –fasta SRR2154279 > ./SRR2154279.fasta). Tandem Repeats Finder (TRFi) analysis and merging of its outputs into groups of ten species were performed as described previously (45). An unfiltered raw TRFi table was obtained by merging all (TRFi) results and was split into two in conversion to Microsoft Excel files (.xlsx). Based on SRA number, data without GENOMIC status reported as the experimental source were removed. Telomere sequence variants known from the literature screen were reduced to eliminate redundancy by removing (i) sequences with <5 nt; (ii) sequences consisting of only 1 letter (e.g. GGGGG); (iii) sequences consisting of 1 letter and multiple copies of other letters (e.g. GCGGG, GGGTGGGG); (iv) sequences in linear plasmids; (v) sequences not with ‘AA’, ‘CC’, ‘GG’, ‘TT’ within the motif (here it meant removal of CACAGA, TACAG and TACACG); (vi) TTCCTC. Iterations of motifs in a raw TRFi table were replaced to match those in the table of selected telomere sequence variants. Matching motifs for each species meeting conditions of at least 5% from the most frequent tandem repeat, or 5% from the first tandem repeat considered as a potential telomere motif were derived for TeloBase. These included calculated ’FTANS’ (First TANdem repeat Share; telomere sequence frequency compared to the tandem repeat with the highest frequency; 100% FTANS has the tandem repeat with the highest frequency in the dataset), and ‘FTELS’ (First TELomere repeat Share; telomere sequence frequency compared to the potential telomere sequence with the highest frequency; 100% FTELS has the first potential telomere sequence in the dataset) for each sequence. Selected telomere sequences used to screen the raw TRFi files are in Supplementary Table S1. Raw TRFi data with highlighted sequences of predicted telomere motifs for species implemented in TeloBase are provided in Additional material (https://www.ceitec.eu/chromatin-molecular-complexes/rg51/tab?tabId=125#TeloBase), or can be downloaded through the link in TeloBase.

### TeloBase construction

The TeloBase was developed in R (version 4.2.2) using the ‘Shiny’ package and is running on a server administered by the Core Facility Bioinformatics, CEITEC Masaryk University, Brno, Czech Republic. Gathered data were processed and organized in CSV files and are used by the database for data retrieval. Taxonomy for newly submitted entries to the database is generated by the same script as described below with the addition that genus/family from the first taxonomic search checks the internal TeloBase taxonomy first to fill in the missing taxonomic ranks before running the second iteration. Tree-based visualisation of telomere sequence distribution in taxa utilizes the ‘Metacoder’ package (46). More detailed description of the data processing steps and of the TeloBase organisation are provided in Additional material and within links in Data Availability section.

### Generation of taxonomy

Taxonomic information for entries was generated by custom-made R script using the ‘taxize’ package (47) that searched through three taxonomic resources, (i) Global Biodiversity Information Facility (GBIF, http://www.gbif.org/), (ii) NCBI (National Center for Biotechnology Information) (48,49) and (iii) TOL (Open Tree of Life) (50), using the name of the organism from the literature or SRA database (NCBI). Lower taxonomic ranks (species to family) were resolved by all three resources in the order of importance GBIF > NCBI > TOL, while higher taxonomic ranks (order to kingdom) used only GBIF information, using the resolved family rank from the first search as the new query. In case that family taxonomic rank remained unknown, GBIF taxonomy from the first iteration was used to fill in the gaps in the higher taxonomic ranks. Data were manually checked for missing or badly implemented information.

### Taxonomic visualisation of telomere sequence distribution

Telomere sequence distribution in taxonomic ranks was created using the TeloBase function for heat tree plot generation. Heat tree plots for selected telomere motif or their combination in specific taxa were blended in Adobe Photoshop CS6.

### Confirmation of new telomere candidates

We used a published TR sequence (GenBank: JQ793887) of *Aspergillus nidulans* as a query in BLASTN (51) with default parameters against several assembled genomes of species belonging to the genus *Aspergillus*. The matching sequences of relevant size and their close proximity (ca. up to 2000 nt) were considered as orthologues. Initially, TR sequences found were further used as queries to search sources (masked assemblies or all model transcripts) of other *Aspergillus* species by BLASTN (51) (default parameters) in MycoCosm genomic resource (52,53) based on taxonomic proximity (54). Orthologues were pairwise aligned using MUSCLE (default settings) in MEGA-X (55) and the loci corresponding to the template regions were compared with the candidate telomere sequences similarly as in (38). TR sequences for species in the family Chrysobalanaceae were identified in WGS SRA Archive (44) (SRR1179646 and SRR1179653) by using BLASTN (51) (default parameters) with TR query from *Caryocar brasiliense* (a close relative to the family Chrysobalanaceae with available genome assembly). Identified TR-like reads were assembled de novo in Geneious Prime 2020.2.5 (https://www.geneious.com). Details about putative TR genes in the genus *Aspergillus* and family Chrysobalanaceae are available in Supplementary Table S2.

### Evaluation of the NGS data contamination

Raw read files of *Palaeopropithecus maximus* sequencing data (SRR1778592, SRA Archive (NCBI) (44)) were uniformly subsampled to 1000 reads using Seqtk sample v1.3 (Li, unpublished, https://github.com/lh3/seqtk) and aligned against Nucleotide DB (NCBI) using BLAST v2.13.0 (51) with the assistance of the NCBI Taxonomy (48,49). The following postprocessing used an in-house script with data filtering by the expected value <1e-15 to get a compact table of taxonomic unit abundance per sample. Additional detection of contaminating sequences in *P. maximus* raw reads utilized BioBloom Tools v2.3.5 (56) with the inclusion of raw sequencing data of *Aspergillus restrictus* (SRR8397711, SRA Archive (NCBI) (44)) as one of the contamination reference sequences.

## Results and discussion

### Collected data of telomere DNA sequences

**Figure 1.**
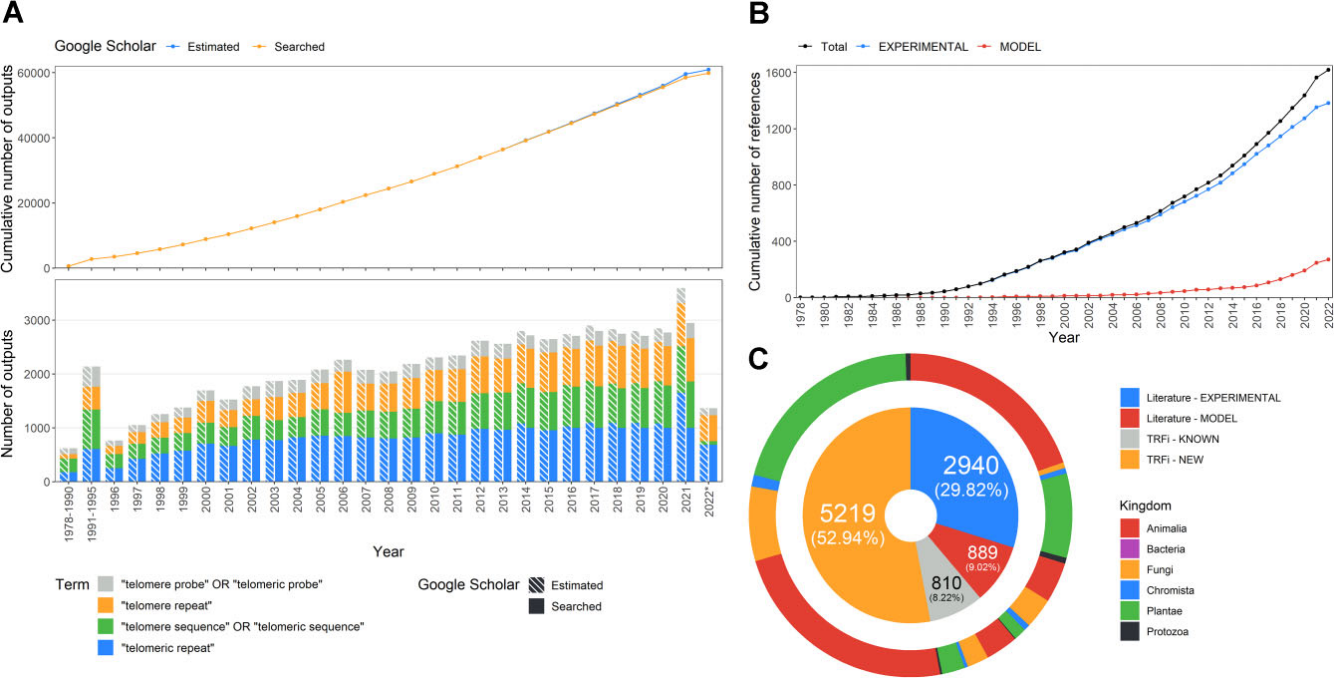
Summary statistics of the data collected in TeloBase. (**A**) Number of literature outputs for selected keywords and the cumulative number of estimated (estimated number of literature outputs by Google Scholar) and searched (actually searched literature outputs) outputs available in Google Scholar versus selected time period; * last search was conducted in September 2022. (**B**) Cumulative number of references added to TeloBase versus the year of publication. EXPERIMENTAL—publications where a telomere sequence was confirmed through ‘wet-lab’ means (first generation sequencing included), MODEL—publications where telomere sequence was confirmed by NGS data analysis or deduced from a putative telomerase RNA template region. (**C**) Nested doughnut chart of species included in TeloBase based on their origin and predicted taxonomic classification. The inner donut chart shows data origin in dataset downloaded from the TeloBase with ‘Filter to one’ option enabled. The outer doughnut chart shows taxonomic distribution of the data within the individual parts of the inner chart by the internal TeloBase taxonomy; TRFi—KNOWN, putative telomere repeats based on tandem repeat finder analysis in species reported in literature; TRFi—NEW, putative telomere repeats based on tandem repeat finder analysis in species reported in this work.

We gathered an extensive collection of data related to telomere sequences using Google Scholar as well as by an additional systematic review of relevant review articles. Google Scholar was chosen as the primary source to find relevant research articles given its capability to search through the full-text of publications (57). Albeit a high number of searched items, reaching almost 60 000 (Figure 1A), many of them corresponded to literature other than peer-reviewed articles (e.g. thesis, books chapters, preprints), especially within later pages of search results. Due to our keyword selection, search results were further diluted with literature dealing with loosely related topics, mostly from cancer research. In years 2014–2021, some of the selected keywords over-reached the Google Scholar hit limit (maximum 1000 results, i.e. 100 pages) (Figure 1A). This could be associated with the popularity of telomeres as a research topic and overall growing research output. Although it is hard to estimate the completeness of our literature screen, we expect that some of the relevant literature might have been missed due to the limited number of keywords as well as search engine specificities (e.g. hit limit, ordering of articles using proprietary algorithm). The same time period (since 2014), when certain keywords were over the hit limit (Figure 1A), corresponded with the increasing number of relevant papers, describing telomere motif composition using NGS data or TR template region (MODEL, Figure 1B). Therefore, further literature screens should focus more on collection of articles dealing with genome assembly. Nevertheless, our literature search discovered 1619 relevant papers (Figure 1B), which was 20 and 26 times higher than references cited in Plant rDNA database (43) and Telomerase database (41), respectively. Within these papers, ‘wet-lab’ techniques (e.g. FISH, hybridization, first generation sequencing techniques; EXPERIMENTAL) were used to describe telomere motifs in 1383 articles, and in 271 articles, NGS data analyses or analyses of TR subunit were applied (MODEL) (Figure 1B).

To increase the number of species with currently known/predicted telomere motifs, we searched for telomere candidates in available raw sequencing data stored in SRA repository (NCBI) (44). Our approach, utilizing Tandem Repeats Finder analysis (TRFi), increased the number of species within the dataset by 136% (5219 species). In comparison, a literature search identified 2940 species with EXPERIMENTAL status and 889 species with only MODEL status (Figure 1C). An additional 810 species from the raw data search matched those from the literature screen (Figure 1C). This additional data can further serve to confirm and validate already known sequences. Based on the internal TeloBase taxonomy, the majority of the species with identified telomere sequences by either approach belong to the kingdoms Animalia and Plantae (Figure 1C).

### The TeloBase database

**Figure 2.**
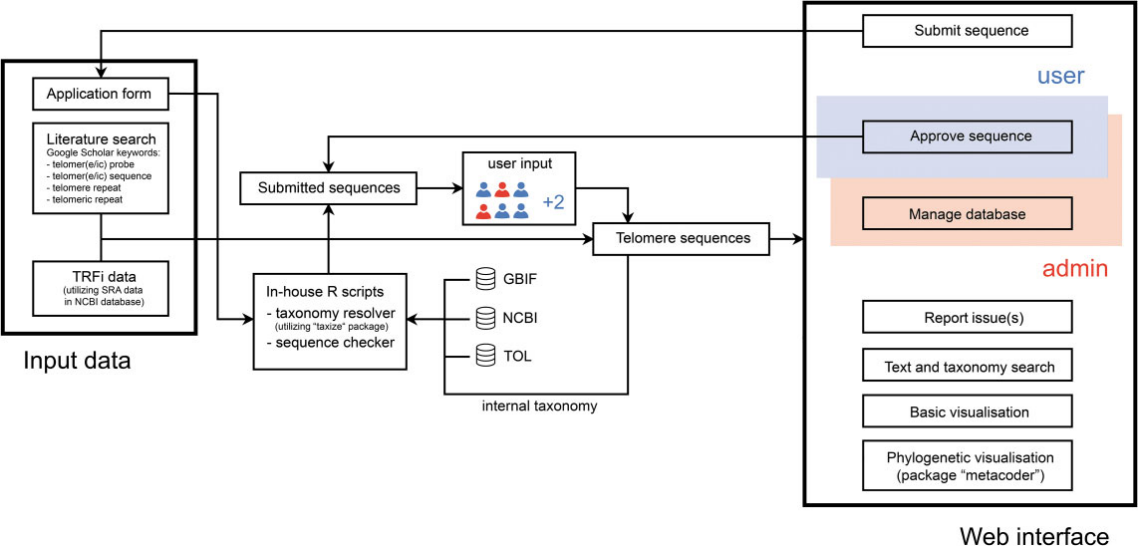
Overview of the TeloBase system. Data was collected from the literature search, NGS data, or can be collected interactively. Application form allows autocompletion of taxonomy information and after submission, submitted telomere sequence phasing is automatically rewritten to conform to one already present within the database (e.g. GGTTAG will be automatically changed to TTAGGG which is already present in the database). Interactively submitted data needs approval from at least two users/administrators before being transferred to the database of collected telomere sequences. Through the web interface, it is possible to submit new sequences, approve newly submitted data, report issues, manage, manipulate and visualize database data.

**Figure 3.**
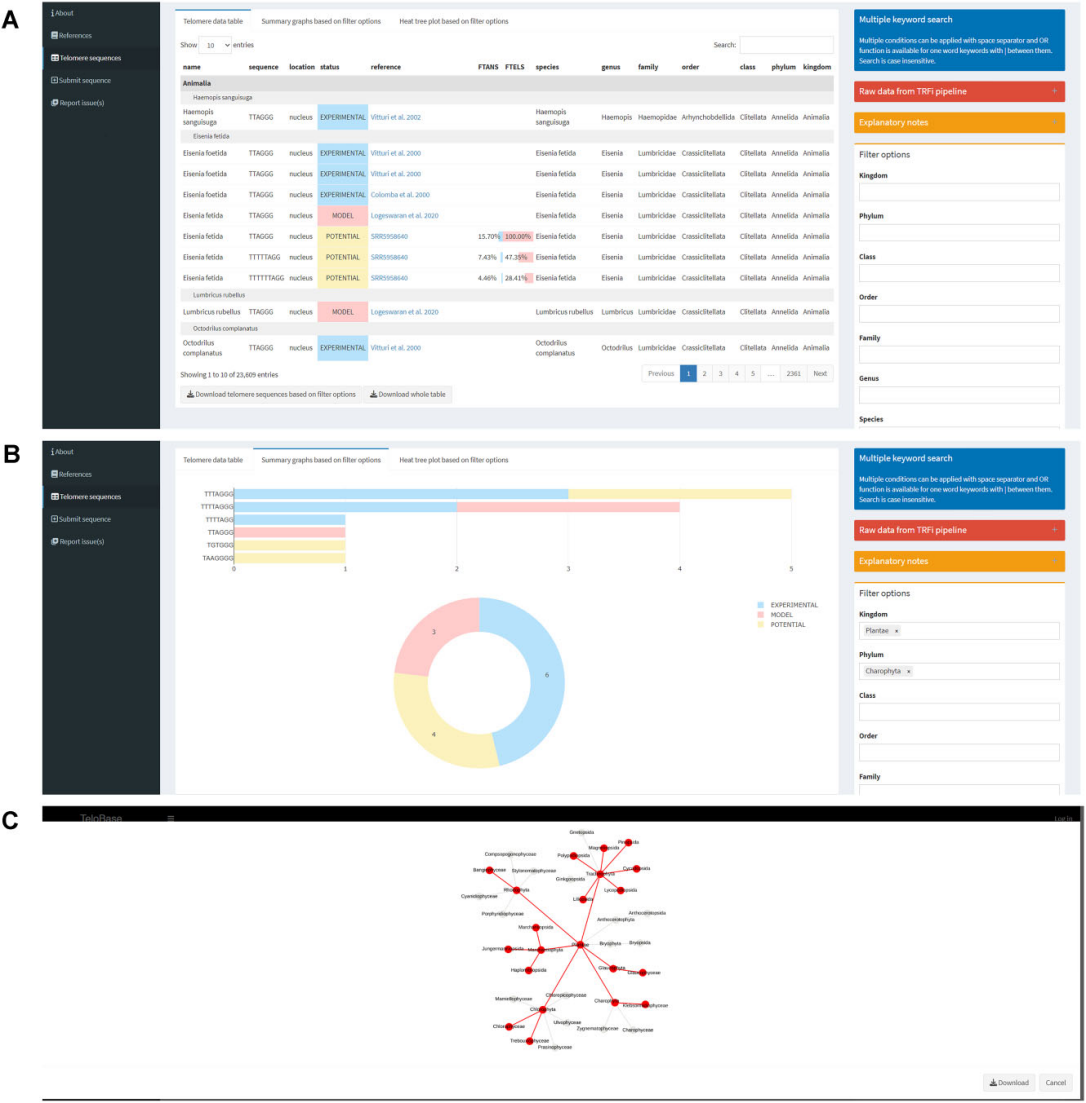
Snapshots of the main TeloBase functionalities within the ‘Telomere sequences’ tab. (**A**) Telomere data table, (**B**) basic summary statistics within selected phylum Charophyta, (**C**) heat tree plot generator highlighting (TTAGGG)_n_ telomere motif within the kingdom Plantae. Although (TTTAGGG)_*n*_ telomere motif is considered a canonical plant telomere sequence, it is evident that also human-type of telomere repeat is present in numerous phyla of plants.

To accommodate the dataset, we developed TeloBase which is available on-line at http://cfb.ceitec.muni.cz/telobase. The homepage shows an overview of data stored within the database and other general information, as well as quick access to available features (e.g. visualisation and plotting of the stored data, application of a new entry, reporting), including those accessible only to registered users and administrators that are connected with data curation. A schematic diagram of the database is shown in Figure 2. Input data, collected either by authors or from newly submitted data, are stored in ‘Telomere data table’, accessible in ‘Telomere sequences’ tab (Figure 3A). Each entry is provided with a name, sequence, location, status, link to the publication or SRA Archive (NCBI) (44), depending on the status of the entry, and taxonomic information. It is necessary to mention that internal TeloBase taxonomy does not serve as a proper source of taxonomic classification *per se*, but applies the state-of-the-art taxonomy databases to categorize the data within the database. Entries with POTENTIAL status (coming from the TRFi analysis only) include information with regard to sequence abundance within the raw TRFi dataset. Data can be filtered based on taxonomy, status and abundance (in case of data with POTENTIAL status). Statistics summarizing the visible data are also provided (Figure 3B). Detailed information about applied terms and features are described in the TeloBase user's manual (see Additional material).

#### Tree-based visualisation of telomere sequences distribution within a respective taxon

TeloBase enables the user to colorize the distribution of selected telomere sequence(s) within the internal taxonomy in the form of heat tree plots (Figure 3C). The plot generation is limited to one colour and, due to computational limits, only two descending taxonomic ranks from the selected taxonomic name are plotted. This function can be beneficial for analysis of potential switches in telomere sequences that are prevalent within some taxonomic groups (1,20). We used this function to create the ‘Atlas of telomere sequences’ from the literature search, which, in a simple form of heat tree plot, demonstrates current knowledge about the diversity of telomere motifs within the tree of life (see Additional material).

#### Application of new telomere sequences and sequence curation

**Figure 4.**
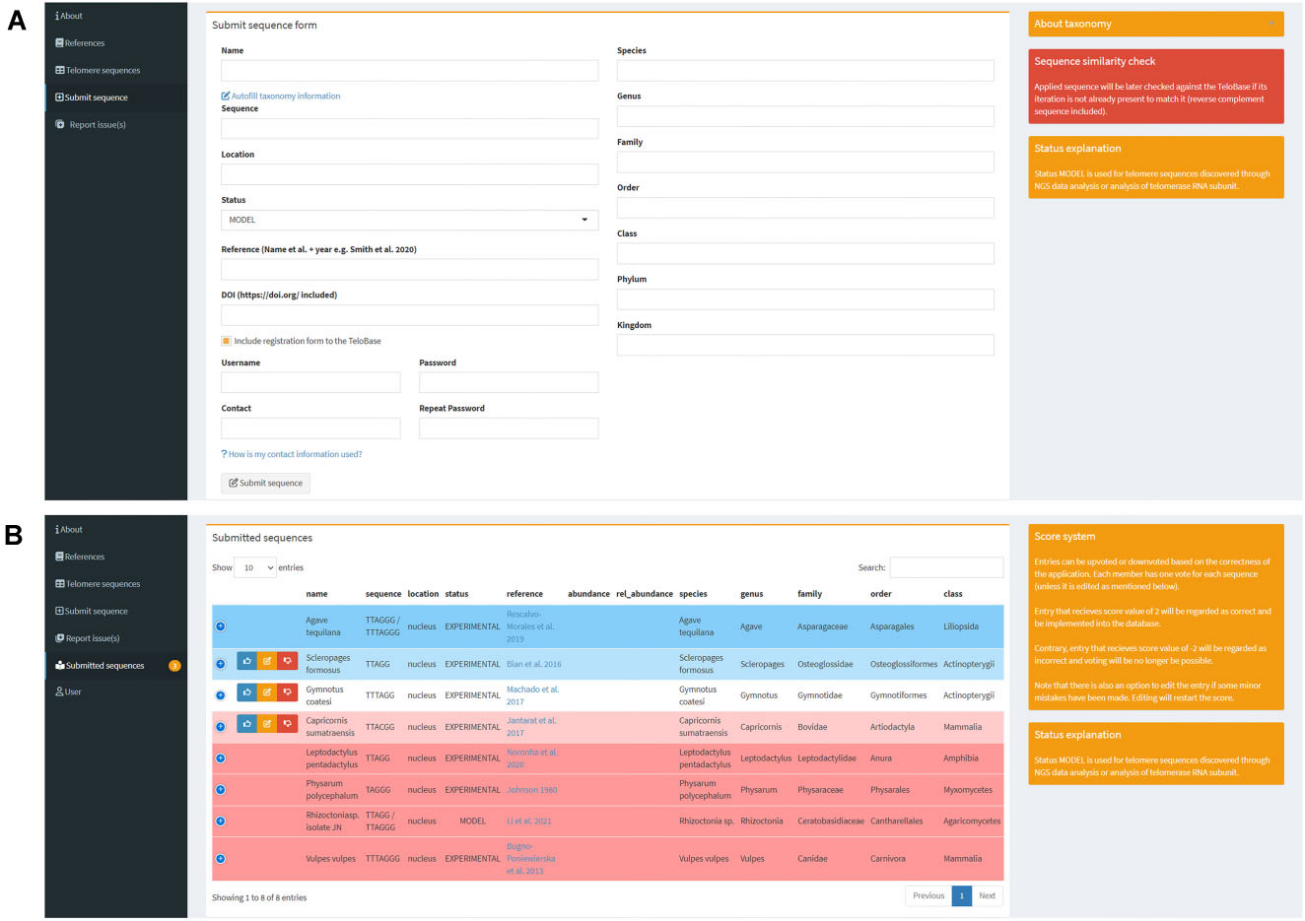
Snapshots of the application process of a new entry. (**A**) Form for sending new entry data and registration located in ‘Submit sequence’ tab, (**B**) ‘Submitted sequences’ tab available to registered users.

Additional entries to the TeloBase are not dependent on administrators and can be added by any user after a peer-review of the submitted entry by registered users. The application form (Figure 4A) requires filling in several boxes, describing the entry (name, sequence, publication background (i.e. DOI and reference) and taxonomy). To speed up the submission process, taxonomic information can be automatically filled based on the internal taxonomy and combination of external taxonomic resources. TeloBase also automatically converts an entered telomere sequence to its iteration that is already present within the database (e.g. ACCCTA to TTAGGG). Contribution to the TeloBase can be connected with the e-mail registration, which is the only way to register, apart from direct invitation from administrators. An e-mail address can be removed from the system later, as its main purpose is just to inform the user once their submission has been accepted and the registration processed.

Registered users are able to curate submitted entries (Figure 4B). Score +2 is required for the entry to be accepted and score −2 locks the application from further modification and voting, leaving it denied (Figures 2 and 4B).

#### Reporting sequence(s)

Visitors can report problematic entries within the ‘Telomere data table’ using a simple form. The report is available for the administrators to process.

### TeloBase brings novel insights into telomere diversity

A good example of TeloBase utility can be demonstrated using species of the genus *Aspergillus*, in which telomere sequences with POTENTIAL status from NGS data analysis showed potentially great diversity between species (see TeloBase). Previous studies identified sequences (TTAGGGTCAACA)_*n*_ in *A. oryzae* from section *Flavi* (58,59) and (TTAGGG)_*n*_ in *A. nidulantes* from section *Nidulantes* (60–62) as telomere motifs. Bioinformatic analyses further showed possible diversity in telomere repeats in section *Nigri* and potentially in section *Terrei* (63), while in sections *Cirumdati*, *Clavati* and *Fumigati*, (TTAGGG)_*n*_ was predicted as a telomere sequence (63–66) (for a detailed list of species and their telomere motifs, see TeloBase). Using the recently published classification of genus *Aspergillus* into subdivisons (54) and by analysing template regions of putative TRs, we significantly expanded current knowledge about telomere diversity within the genus *Aspergillus* (Table 1). (TATTAGGG)_*n*_ found in *A. taichungensis* from section *Candidi*, and (TTATTAGGG)_*n*_ discovered in *A. transcarpathicus* from section *Cervini*, are especially interesting as they represent novel telomere motif variants that have not yet been observed in nature (see TeloBase).

**Table 1.** Selected *Aspergillus* species with identified motifs by TRFi and by analysis of the putative TR template region

Subgenus	Section	Series	Species	POTENTIAL motif	Probable motif	Probable TR template region (5′-3′)
***Circumdati***	*Candidi*	*Candidi*	*A. taichungensis*	CCTAAT	CCCTAATA*	ggaatctg[(ACCCTAAT)ACCCTA]gtcg
	*Nigri*	*Nigri*	*A. neoniger*	CCCTAAAA	CCCTAAAA	ggaact[(AAACCCTA)A]tagacaccaat
		*Carbonarii*	*A. carbonarius*	CCCTAATAAA / CCCTAAA	CCCTAAA	ggaactt[(AACCCTA)A]tacacaccaat
		*Heteromorphi*	*A. ellipticus*	CCCTAAA	CCCTAAA	ggaatct[(AACCCTA)AA]actcttagtc
		*Japonici*	*A. floridensis*	various	CCCTAATGTAA	ggagtc[(TAACCCTAATG)TAACCC]cgt
	*Terrei*	*Terrei*	*A. terreus*	CCCTAAA	CCCTAAA	ggaatct[(AACCCTA)A]gaataacctgg
		*Nivei*	*A. allahabadi*	CCCTAAA	CCCTAAA	ggaatct[(AACCCTA)A]ggaaacccttg
	*Janorum*	*Janorum*	*A. janus*	CCCTAATAAA	CCCTAATAAA	ggaatct[(AACCCTAATA)A]cccatcgt
	*Circumdati*	*Circumdati*	*A. affinis*	CCCTAATGTAA	CCCTAATGTAA	agagct[(TAACCCTAATG)TAACCC]agt
		*Steyniorum*	*A. pulvericola*	various	CCCTAATGTAA	ggaatc[(TAACCCTAATG)TAACCC]agt
		*Sclerotiorum*	*A. sclerotiorum*	CCCTAA	CCCTAA	ggaacc[(TAACCC)TAA]gtaccccaag
		*Sclerotiorum*	*A. subramanianii*	CCCTAA(A)	CCCTAA(A)	ggaacc[TAACCCTAA]gtaccccaagtc
	*Flavi*	*Flavi*	*A. parasiticus*	CCCTAATGTTGA	CCCTAATGTTGA	ggaatc[(TGACCCTAATGT)TGACC]aag
		*Flavi*	*A. novoparasiticus*	CCCCTAA / CCCTAATGTTGA	CCCTAATGTTGA	ggaatc[(TGACCCTAATGT)TGACC]aag
		*Kitamyces*	*A. caelatus*	CCCTAATGTTGA	CCCTAATGTTGA	ggaatc[(TGACCCTAATGT)TGACC]aag
		*Bertholletiarum*	*A. bertholletiae*	CCCTAATGTTGA	CCCTAATGTTGA	agaatc[(TGACCCTAATGT)TGACC]aag
		*Nomiarum*	*A. pseudonomius*	CCCTAATGTTGA	CCCTAATGTTGA	ggaatc[(TGACCCTAATGT)TGACC]aag
***Nidulantes***	*Nidulantes*	*Stellati*	*A. astellatus*	CCCTAAAA	CCCTAAAA	ggaact[(AAACCCTA)A]tagacaccaat
		*Stellati*	*A. filifer*	CCCTAA	CCCTAA	ggaatc[(TAACCC)TAA]gccaactttgt
		*Aurantiobrunnei*	*A. aurantiobrunneus*	CCCTAA	CCCTAA	ggaatc[(TAACCC)TAA]gacaaattcgt
	*Usti*	*Calidousti*	*A. keveii*	CCCTAA	CCCTAA	ggaatc[(TAACCC)TAA]tgcacttcgtc
	*Bispori*	*Bispori*	*A. bisporus*	CCCTAA(A)	CCCTAA(A)	ggaatc[TAACCCTAA]tgtatctttgtc
	*Ochraceorosei*	*Ochraceorosei*	*A. ochraceoroseus*	CCCTAA	CCCTAA	ggaatc[(TAACCC)TAA]tgtacctttgt
***Fumigati***	*Fumigati*	*Fumigati*	*A. fischeri*	CCCTAA	CCCTAA	ggaatc[(TAACCC)TAACC]tagtcggtt
	*Cervini*	*Cervini*	*A. parvulus*	CCCTAATAAA	CCCTAATAAA	ggaatct[(AACCCTAATA)A]cctagtcg
		*Cervini*	*A. transcarpathicus*	CCCTCA**	CCCTAATAA*	ggaatc[(TAACCCTAA)TAACC]tagtcg
***Aspergillus***	*Restricti*	*Restricti*	*A. restrictus*	CCCCAATAA	CCCCAATAA	ggaac[(ATAACCCCA)ATAACC]atgg
***Polypaecilum***	*Polypaecilum*	*Polypaecilum*	*A. insolitus*	CCCTAAA	CCCTAAA	ggaacc[(TAAACCC)TAA]taccctagtc

Classification of genus *Aspergillus* into subdivisons was based on (54). Probable motif with POTENTIAL status was taken from the TeloBase. Parentheses highlights putative telomere motif and square brackets include the putative annealing part of the template region. Detailed information about putative TRs from which template regions were used to predict probable telomere motifs can be found in Supplementary Table S2.

*Novel motif.

**Probable motif is present in raw TRFi data (see Additional material).

Similarly, we were able to reveal a new telomere sequence candidate (TTTATTAGGG)_*n*_ for vascular plants in species belonging to the family Chrysobalanaceae, a tropical family of woody plants often ecologically dominant in the Neotropics (67) (Table 2). This telomere motif is not novel for plants as it was previously described in species of the genus *Galdieria* (red algae) (19,68). Bioinformatic analysis of TRs discovered in *Hirtella physophora* and *Licania alba* confirmed the presence of (TTTATTAGGG)_*n*_ in their template regions (Table 2). More detailed information about putative TR genes in the genus *Aspergillus* and the family Chrysobalanaceae can be found in Supplementary Table S2.

**Table 2.** Species from family Chrysobalanaceae with identified motifs by TRFi and motif prediction based on the putative TR template region

Species	POTENTIAL motif	Probable motif	Probable TR template region (5′-3′)
*Couepia guianensis*	CCCTAATAAA	NA	NA
*Hirtella physophora*	CCCTAATAAA	CCCTAATAAA	gtaacct[(AACCCTAATA)AACCCT]tagcgc
*Hirtella racemosa*	CCCTAATAAA	NA	NA
*Chrysobalanus icaco*	CCCTAATAAA	NA	NA
*Licania alba*	CCCTAATAAA	CCCTAATAAA	gtaaccg[(AACCCTAATA)AACCCT]tagcac
*Licania sprucei*	CCCTAATAAA	NA	NA
*Parinari campestris*	CCCTAATAAA	NA	NA

Probable motif with POTENTIAL status was taken from the TeloBase. Parentheses highlights a putative telomere motif and square brackets include the putative annealing part of the template region. Detailed information about putative TRs, from which template regions were used to predict probable telomere motifs, can be found in Supplementary Table S2.

### Telomere sequences with POTENTIAL status require confirmation

It is apparent that screening for tandem repeats in NGS data can help to unravel telomere sequence composition as shown above and as previously proven for other species (20,36,40). However, POTENTIAL status of such sequences needs to be emphasised, as their presence in NGS datasets does not guarantee their localization in the telomere region, the sequencing of which may also fail to be enriched, as we demonstrated for *A. floridensis and A. pulvericola* (Table 1). It is also possible that telomere sequences with POTENTIAL status can, in some cases, just be contaminating reads of other species within the sequencing data. For example, tandem repeat analysis of *Palaeopropithecus maximus* sequencing data (SRR1778592, SRA Archive (NCBI) (44)) showed a high abundance of the (TTATTGGGG)_*n*_ motif, followed by (TTTAGGG)_*n*_ reaching around 40% FTELS (see TeloBase). However, it is expected that the *P. maximus* telomere sequence corresponds to (TTAGGG)_*n*_, as in other vertebrates including closely related *P. ingens* where the ‘vertebrate-like’ telomere sequence represents the only predicted POTENTIAL motif (see TeloBase). Given that *P. maximus* belongs to the extinct giant lemur genera, and the data originated from an ancient femur bone (69), we suspected possible contamination of the stored subfossil material. A query of the (TTATTGGGG)_*n*_ sequence in TeloBase revealed that this sequence was reported with the MODEL status as telomeres of fig wasps and parasitoid wasps (35,37), and with the POTENTIAL status in *A. restrictus* in which we bioinformatically confirmed (TTATTGGGG)_*n*_ as the telomere motif (Table 1). *A. restrictus* or possibly other *Aspergillus* species from the *Restricti* section (70), where the same telomere sequence can be expected, seemed to be the likely contaminants, given their xerophilic nature that enables them to colonize even museum repositories under controlled conditions (71).

In agreement with our hypothesis, analysis of the sequencing data against Nucleotide DB (NCBI) confirmed the presence of foreign reads belonging to *Aspergillus* genera (Supplementary Table S3). Categorizing sequences with BioBloom tools (56), with inclusion of *A. restrictus* sequencing data (SRR8397711, SRA Archive (NCBI) (44)), revealed a unique 1.4% *k*-mer match with *A. restrictus* (Supplementary Table S4). Unexpectedly high abundance of (TTTAGGG)_n_ might also be connected with *Aspergillus* contamination given that this sequence was predicted as a telomere motif with POTENTIAL status in several species of this genera (Table 1 and TeloBase). Given the surprisingly low abundance of foreign reads and yet clear abundance of telomere-like repeats in TRFi analysis that do not match the expected ‘vertebrate-like’ motif, we suggest that TRFi analysis in connection with the TeloBase repository might represent a useful additional hint when looking for potential contaminants in NGS data.

## Concluding remarks

We have gathered a comprehensive dataset of telomere sequences from literature and NGS data searches, which were implemented in the TeloBase database. TeloBase allows interactive manipulation and visualisation of the data, as well as simple application and community curation of new entries. With these features, we envisage that TeloBase represents a solid and viable source of information about telomere motif diversity and telomere evolution for the future, which is expected to bring increasingly more knowledge, especially due to the rapidly accelerating amount of available sequencing data.

## Supplementary Material

gkad672_Supplemental_Files

## Data Availability

TeloBase is available at http://cfb.ceitec.muni.cz/telobase. Additional material (e.g. raw data from TRFi analysis with highlighted candidate sequences, Atlas of telomere sequences, TeloBase user manual) as well as a link to the database are available at https://www.ceitec.eu/chromatin-molecularcomplexes/rg51/tab?tabId=125#TeloBase. Scripts for taxonomy generation and for tree-based visualisation that are implemented in TeloBase are available at https://github.com/mlyc93/TeloBase (https://doi.org/10.5281/zenodo.8207504).
