# Supplementary material for: TeloBase: a community-curated database of telomere sequences across the tree of life
Source: Nucleic Acids Res. 2023 Aug 21;52(D1):D311–21. doi: 10.1093/nar/gkad672 (PMC10767889; doi:10.1093/nar/gkad672)
Supplement: gkad672_Supplemental_Files [file gkad672_supplemental_files.zip › Supplementary Table S3 - contamination of NGS using Nucleotide DB.docx]

Supplementary Table S3 Contamination of *Palaeopropithecus maximus* SRA data (SRR1778592) based on Nucleotide DB (NCBI)

| Species | Abundance (%) |
| --- | --- |
| Aspergillus fischeri NRRL 181 | 0.2 |
| Aspergillus | 0.2 |
| Aspergillus | 0.2 |
| uncultured fungus | 0.2 |
| Amycolatopsis methanolica 239 | 0.1 |
| Annulohypoxylon stygium | 0.1 |
| Aspergillus clavatus | 0.1 |
| Aspergillus clavatus NRRL 1 | 0.1 |
| Aspergillus fumigatus | 0.1 |
| Aspergillus fumigatus Af293 | 0.1 |
| Other species | 1.9 |
| Insignificant hits | 96.7 |

Contaminating species were detected for randomly subsampled 1000 reads with valid hits having E-value < 1E-05.
