# Supplementary material for: TeloBase: a community-curated database of telomere sequences across the tree of life
Source: Nucleic Acids Res. 2023 Aug 21;52(D1):D311–21. doi: 10.1093/nar/gkad672 (PMC10767889; doi:10.1093/nar/gkad672)
Supplement: gkad672_Supplemental_Files [file gkad672_supplemental_files.zip › Supplementary Table S4 - contamination of NGS using BioBloom Tools.docx]

Supplementary Table S4 Contamination of *Palaeopropithecus maximus* SRA data (SRR1778592) based on BioBloom tools

| Species | Abundance (%) |
| --- | --- |
| Aspergillus restrictus | 1.4 |
| human_38 | 0.2 |
| mouse | 0.1 |
| Arabidopsis thaliana | 0.0 |
| Drosophila melanogaster | 0.0 |
| Caenorhabditis elegans | 0.0 |
| yeast | 0.0 |
| Multiple Genomes | 0.8 |
| No Match | 97.5 |

*Aspergillus restrictus* SRA data (SRR8397711) was included as one of the references for the contamination detection.
